# Evaluation of loop-mediated isothermal amplification (LAMP) assay for detection of five bacterial periprosthetic joint infection

**DOI:** 10.1017/ash.2025.158

**Published:** 2025-09-03

**Authors:** Woong Sik Jang, Ji Hoon Bae, Min-Chul Cho, Chae Seung Lim

**Affiliations:** * Department of Laboratory Medicine, Orthopaedic Surgery Korea University Guro HospitalKorea University College of Medicine, Seoul, Republic of Korea

**Keywords:** bacteria, periprosthetic joint infection, diagnosis, multiplex, loop-mediated isothermal amplification

## Abstract

**Objectives:** Periprosthetic joint infection (PJI) is one of the most serious and debilitating complications that can occur after total joint arthroplasty. Therefore, early diagnosis and appropriate treatment are important for a good prognosis. Recently, molecular diagnostic methods have been widely used to detect the causative microorganisms of PJI sensitively and rapidly. The Multiplex Loop- Mediated Isothermal Amplification (LAMP) method is faster and easier to perform compared to polymerase chain reaction (PCR)-based assays. Therefore, this study developed a multiplex LAMP assay for diagnosing bacterial PJI using LAMP technology and evaluated its analytical and clinical performance. **Methods:** We developed a multiplex LAMP assay for the detection of five bacteria: Staphylococcus aureus, Staphylococcus epidermidis, Streptococcus agalactiae, Pseudomonas aeruginosa, and Escherichia coli, frequently observed to be the causative agents of PJI. The method limit of detection (LOD) and cross-reactivity were determined by spiking standard strains into the joint synovial fluid. The LOD of the multiplex LAMP assay was compared with that of a quantitative real-time PCR (qPCR) assay. Clinical performance was evaluated using 20 joint synovial fluid samples collected from patients suspected of having bacterial PJI. **Results:** The LOD of the gram-positive bacterial multiplex LAMP assay and qPCR were 10^5^/10^4^ CFU/mL, 10^3^/10^3^ CFU/mL, and 10^5^/10^4^ CFU/mL against *S. agalactiae*, *S. epidermidis,* and *S. aureus*, respectively. For *P. aeruginosa* and *E. coli*, the LOD of the multiplex LAMP and qPCR assays were 10^5^/10^4^ and 10^6^/10^4^ CFU/mL, respectively. The multiplex LAMP assay detects target bacteria without cross-reacting with other bacteria, and exhibited 100% sensitivity and specificity in clinical performance evaluation. **Conclusions:** This multiplex LAMP assay can rapidly detect five high-prevalence bacterial species causing bacterial PJI, with excellent sensitivity and specificity, in less than 1 h, and it may be useful for the early diagnosis of PJI.

